# Assembly Strategy and Performance Evaluation of Flexible Thermoelectric Devices

**DOI:** 10.1002/advs.201900584

**Published:** 2019-05-22

**Authors:** Dawei Qu, Xin Li, Hanfu Wang, Guangming Chen

**Affiliations:** ^1^ College of Materials Science and Engineering Shenzhen University Shenzhen 518055 P. R. China; ^2^ Beijing Key Laboratory of Clothing Materials R&D and Assessments Beijing Engineering Research Center of Textile Nanofiber School of Materials Science and Engineering Beijing Institute of Fashion Technology Beijing 100029 P. R. China; ^3^ National Center for Nanoscience and Technology of China Beijing 100190 P. R. China

**Keywords:** assembly strategies, flexible devices, performance evaluation, thermoelectric

## Abstract

Although organic and composite thermoelectric (TE) materials have witnessed explosive developments in the past five years, the research of flexible TE devices is rather limited. In particular, their assembly strategies and device performance reported in the literature cannot be directly compared, due to a variety of deviances including p‐ and n‐type component materials, shape and dimensions of p‐n flexible films, and applied temperature gradient (Δ*T*). Here, three types of assembly strategies for flexible TE devices, that is, serial, folding, and stacking, are compared by fixing the corresponding experimental parameters. Furthermore, a convenient and general method to evaluate the flexible device performance (FDP) is put forward, that is, $\mathrm{FDP}\;\;=\;\;\frac{P\max}{m\Delta TN}$, where the maximum output power (*P*_max_) is divided by product mass (*m*), Δ*T*, and pair number of p‐n couples (*N*). The FDPs for the present serial, folding, and stacking devices are 11.13, 8.87, and 0.05 nW g^−1^ K^−1^, respectively, confirming that the serial configuration is the best among the three strategies for flexible device fabrication. The preliminary evaluation method proposed herein will pave the way for a design strategy of flexible TE devices and speed up their applications in waste‐heat harvesting, e‐skin, wearable electronics, etc.

In the past five years, organic thermoelectric (TE) materials, especially organic polymers and their composites, have witnessed explosive developments.^1^, ^2^, ^3^, ^4^, ^5^, ^6^ One main reason lies in the high flexibility of the TE devices, which can meet the severe requirements for versatile applications in complex environments to harvest waste heat and generate electricity. The TE performance of these materials are generally evaluated by a dimensionless figure of merit (*ZT*), $ZT\;\;=\;\;\frac{S^{2}\sigma }{k}\;T$, where *S*, σ, *k*, and *T* stand for the Seebeck coefficient or thermopower, the electrical conductivity, the thermal conductivity, and the absolute temperature, respectively. In cases of low thermal conductivities for the neat polymers and their composite materials, their TE performance is always evaluated by power factor (PF = *S* ^2^σ).^7^, ^8^, ^9^, ^10^, ^11^, ^12^, ^13^ Very regretfully, the performance evaluation for the flexible TE devices is confused in the available literatures. Indeed, no general and regular standard has been effectively set up so far.

To date, several design strategies have been developed to achieve flexible TE devices by assembling flexible films,^14^, ^15^, ^16^ yarns,^17^, ^18^, ^19^, ^20^ and printing techniques.^21^, ^22^, ^23^, ^24^ Here, we focus on the film assembly configuration, since this is the most extensively studied one till now. In sharp contrast with the common π‐type configuration of inorganic TE devices consisting of alternating p‐ and n‐type legs (**Scheme** 1a), the assembly configurations for the flexible film devices are complex (b–d). In essential, the assembly modes can be divided into three major strategies, namely, (b) serial, (c) folding, and (d) stacking routes. In the serial‐type devices (Scheme 1b), rectangular films of the p‐ and n‐type units are interconnected by electrically conductive metal in series.^25^, ^26^, ^27^ In Scheme 1c, the folding‐type devices are achieved by rolling the continuous flexible film of alternating p‐n units on insulate plastic membrane.^27^ The stacking devices (Scheme 1d) are developed by stacking the multilayered films of p‐ and n‐type TE constituents intercalated by insulate layers.^28^, ^29^, ^30^ Regretfully, it is impossible to make direct comparison and effectively evaluate the assemble strategies among these flexible TE devices, since various parameters defining the device output performance are different, for example, the materials' TE performance of the p‐ and n‐unit films, the film dimensions (length, width, and thickness), the number of p‐n couples (*N*), and even the applied temperature gradients between the hot end and the cold end (Δ*T* = *T*_hot_ − *T*_cold_). Therefore, a clear comparison of rational design of assembly strategies is strongly desired for the flexible TE devices. Furthermore, a general standard or method to effectively evaluate the device TE performance is greatly expected, which is vital for further in‐depth research and wide applications of TE materials.

**Scheme 1:**
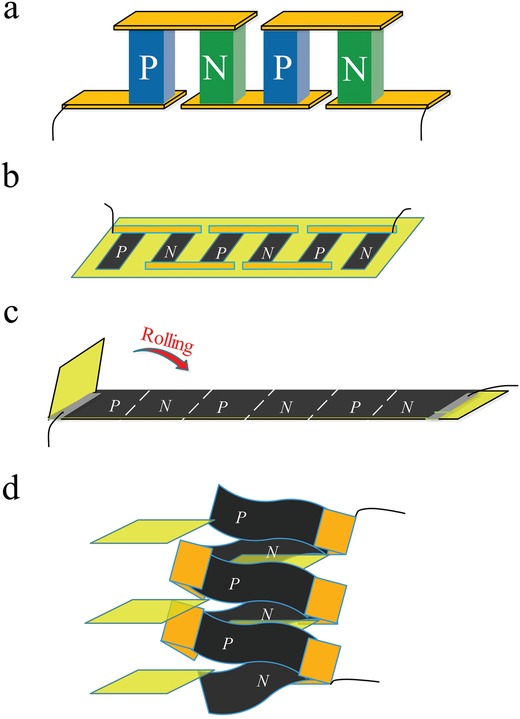
Common assembly styles of a) inorganic π‐configuration TE devices consisting of alternating p‐ and n‐type legs, and b–d) organic flexible devices made up of flexible films, including serial, folding, and stacking modes.

In this study, we report a comparison of the three main assembly strategies for flexible TE devices (serial, stacking, and folding), and put forward a convenient and general evaluation method for flexible device performance (FDP). First, p‐ and n‐type flexible TE films were prepared by chemically doping single‐walled carbon nanotubes (SWCNTs) films by sodium dodecyl benzene sulfonate (SDBS) and polyethyleneimine (PEI), respectively. Then, the flexible devices were fabricated by the three types of configurations, and the output powers were measured. Moreover, we proposed a convenient and general formula to compare different assembling modes using FDP, defined by the following Equation (1)

(1) $$\mathrm{FDP}\;\;=\;\;\frac{P\max}{m\Delta TN}$$

where *P*_max_ and *m* stand for the maximum output power and the device mass, respectively. Finally, the FDPs for the present work were calculated, showing that the serial device is superior to the stacking and folding ones, with an FDP of 11.13 nW g^−1^ K^−1^.

Here, the p‐ and n‐type SWCNT films were prepared by drop‐casting SDBS and PEI aqueous solutions on flexible films of the precursor of acid‐doped SWCNTs (Figure S1, Supporting Information), respectively. Their morphology, structure, and TE property are shown in **Figure** 1. Figure 1A presents the surface morphology for (a) acid‐doped SWCNT, (b) p‐ and (c) n‐type chemically doped SWCNT films by 1 wt% SDBS or PEI solution, using field‐emission scanning electron microscopic (FESEM) observations. Continuous SWCNT bundles are distinct (a), while the SWCNT bundles become vague and rough in (b) and (c) owing to the surface wrapping by the organic SDBS or PEI. Further increase of SDBS or PEI loading leads to thicker coating layers (Figure S2, Supporting Information). Figure S3 (Supporting Information) shows (a) the Raman spectra and (b) the radial breathing mode (RMB) profiles. All display a strong band at ≈1594 cm^−1^ (G band) and a very weak band at ≈1348 cm^−1^ (D band), confirming that after being organically chemical doped by either SDBS or PEI, the high crystallinity of the pristine SWCNT is well reserved, and no obvious defects are introduced. Additionally, the distinct change of RMB, characteristic of an out‐of‐plane stretching phonon mode, reveals that the carbon atoms moving coherently in the radial direction are hindered after the SDBS or PEI doping.^29^ Moreover, X‐ray photoemission spectroscopy (XPS) further verify the doping effectiveness (Figure 1B). Besides the strong peak at 284.4 eV of C1s, the occurrence of the weak O1s peak (532.6 eV) results from the acid‐doping (HNO_3_/H_2_SO_4_), the surface defects, and the doping agent of SDBS and PEI. Similarly, the N1s peak at 398.9 eV for the PEI‐doped SWCNT is due to the existence of PEI molecules. In addition, the presence of the two peaks at 1071.6 (Na1s) and 168.5 eV (S2p) results from the doping agent of SDBS. In addition, the Fourier transform infrared (FTIR) spectra (Figure S4, Supporting Information) confirm the effective chemical doping by SDBS and PEI as well.

**Figure 1:**
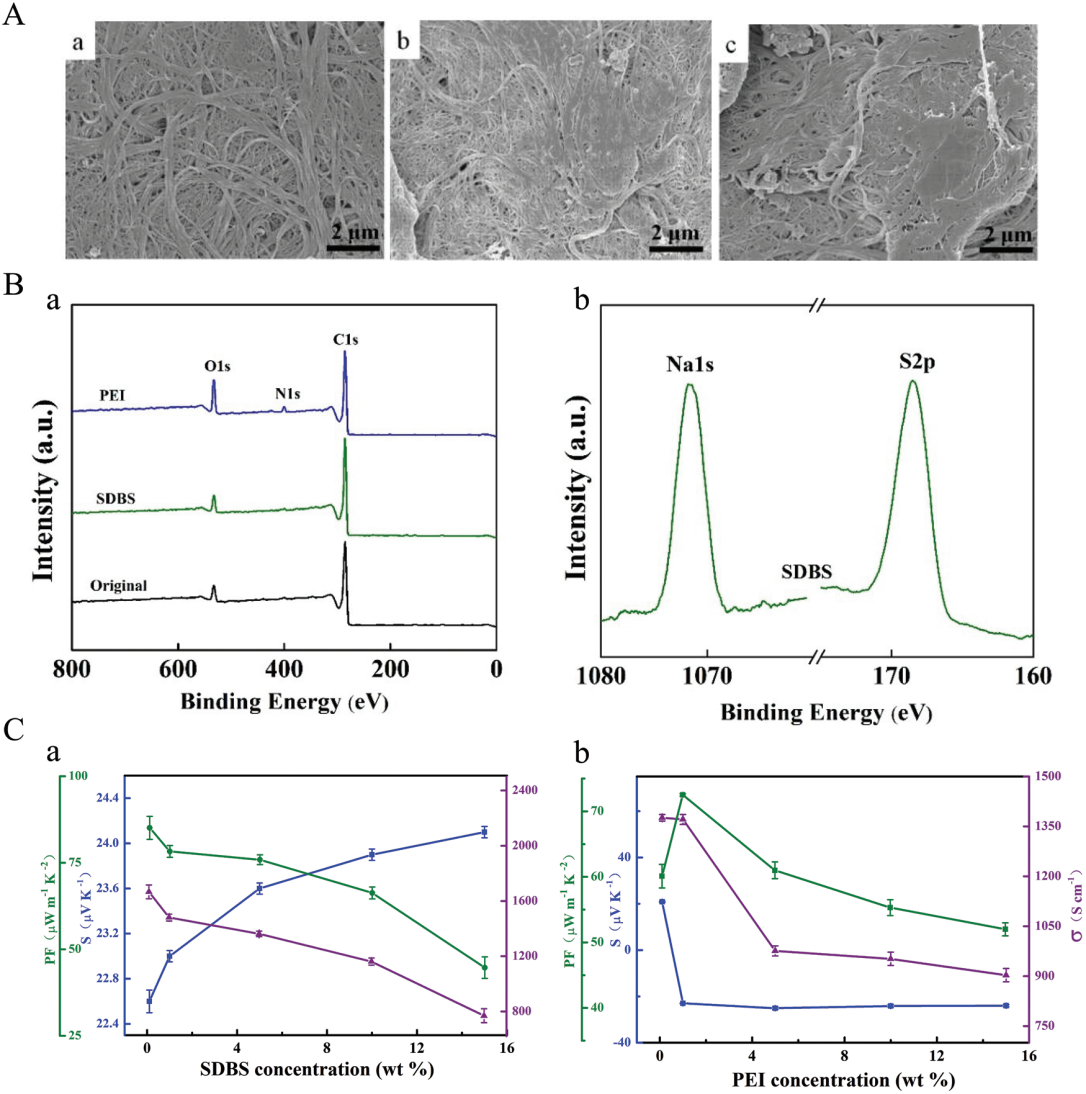
A) Morphological observations by FESEM images of a) acid‐doped SWCNT and b) 1 wt% SDBS or c) 1 wt% PEI doped SWCNT, B) XPS spectra, and C) TE performance at room temperature for the pristine acid‐doped SWCNT, and the SDBS or PEI‐doped SWCNTs.

Figure 1C displays the effect of doping agent (SDBS or PEI) concentration on the composite TE performance at room temperature. Because of the formation of a thick insulating layer of SDBS or PEI coating on SWCNT surfaces, the electrical conductivities of the doped SWCNT films reduce with the increase of SDBS or PEI concentration. Transferring of majority carriers from holes to electrons, due to electron donation from amine‐rich PEI molecules to SWCNT surfaces, also contributes to the reduction of electrical conductivities for PEI doping. In contrast, with increased SDBS concentration, the Seebeck coefficient increases monotonically from 18.7 µV K^−1^ (acid‐doped SWCNT) to 24.2 µV K^−1^ (15 wt% SDBS‐doped SWCNT). The increased Seebeck coefficient may be due to the interfacial barriers that prevent low‐energy carrier from transporting across the interfaces. As a result, the power factor gradually reduces with increased SDBS concentration. As for PEI doping, the Seebeck coefficient decreases significantly from positive to negative even at low PEI concentration of 0.1 wt%, confirming the high effectiveness for the n‐type doping (electron donation) and a successful p‐ to n‐type transition. As a consequence, after the SDBS or PEI treatments, p‐ and n‐type SWCNT films have been achieved. With further increase of the concentration, the Seebeck coefficient for the PEI‐doped SWCNT is almost independent of PEI concentration, being around −24 µV K^−1^. In order to match the Seebeck coefficients between p‐ and n‐type constituents, here, 1 wt% SDBS‐doped SWCNT with a Seebeck coefficient of 22.6 ± 0.1 µV K^−1^ and 1 wt% PEI‐treated SWCNT with −23.0 ± 0.1 µV K^−1^ are chosen as the p‐ and n‐type units for device fabrications. The corresponding electrical conductivities and power factors for the p‐ and n‐type SWCNTs are 1481 ± 25 S cm^−1^ and 78.3 ± 1.7 µW^−1^ m^−1^ K^−2^, 1371 ± 15 S cm^−1^ and 72.5 ± 0.2 µW^−1^ m^−1^ K^−2^, respectively.

In order to gain insights into the assembly strategy for flexible devices, **Figure** 2 displays a schematic illustration to compare the three different design configurations, namely, (a) serial, (b) folding, and (c) stacking. For the serial device (a), three pairs of p‐ and n‐type SWCNT films are pasted on polyimide substrate and electrically connected by Cu foil in series. Then, they are sealed with polyimide tape encapsulation. To achieve a smaller size dimension, the serial device is folded into zigzag shape, and finally encapsulated by polyimide tape. As for the folding configuration (b), the acid‐doped SWCNT film is first drop‐casted by SDBS and PEI aqueous solutions alternatively with the same intervals, resulting in a continuous film consisting of separated p‐ and n‐type units. Note that coverslips are employed to ensure effective doping differently from the adjacent sections. Finally, the folding device is completed by rolling and encapsulation with polyimide tape. In the stacking device (c), three p‐n couples are multilayered stacked, where polyimide films are inserted between adjacent p‐ and n‐type films to avoid any direct contact electrically. The continuous electrical transport is ensured by the Cu foils at the two ends. Then, the device is encapsulated by polyimide tape. More information on the details of the device fabrication can be seen in the Experimental Section. Evidently, all of these three devices obtained herein are quite similar in outer appearance, as shown in Figure S5 (Supporting Information). Moreover, Figure S6 (Supporting Information) clearly shows that the TE device is flexible and easy to bend, which is crucial to ensure close contact with the surfaces of heat resources and make optimum use of waste heat.

**Figure 2:**
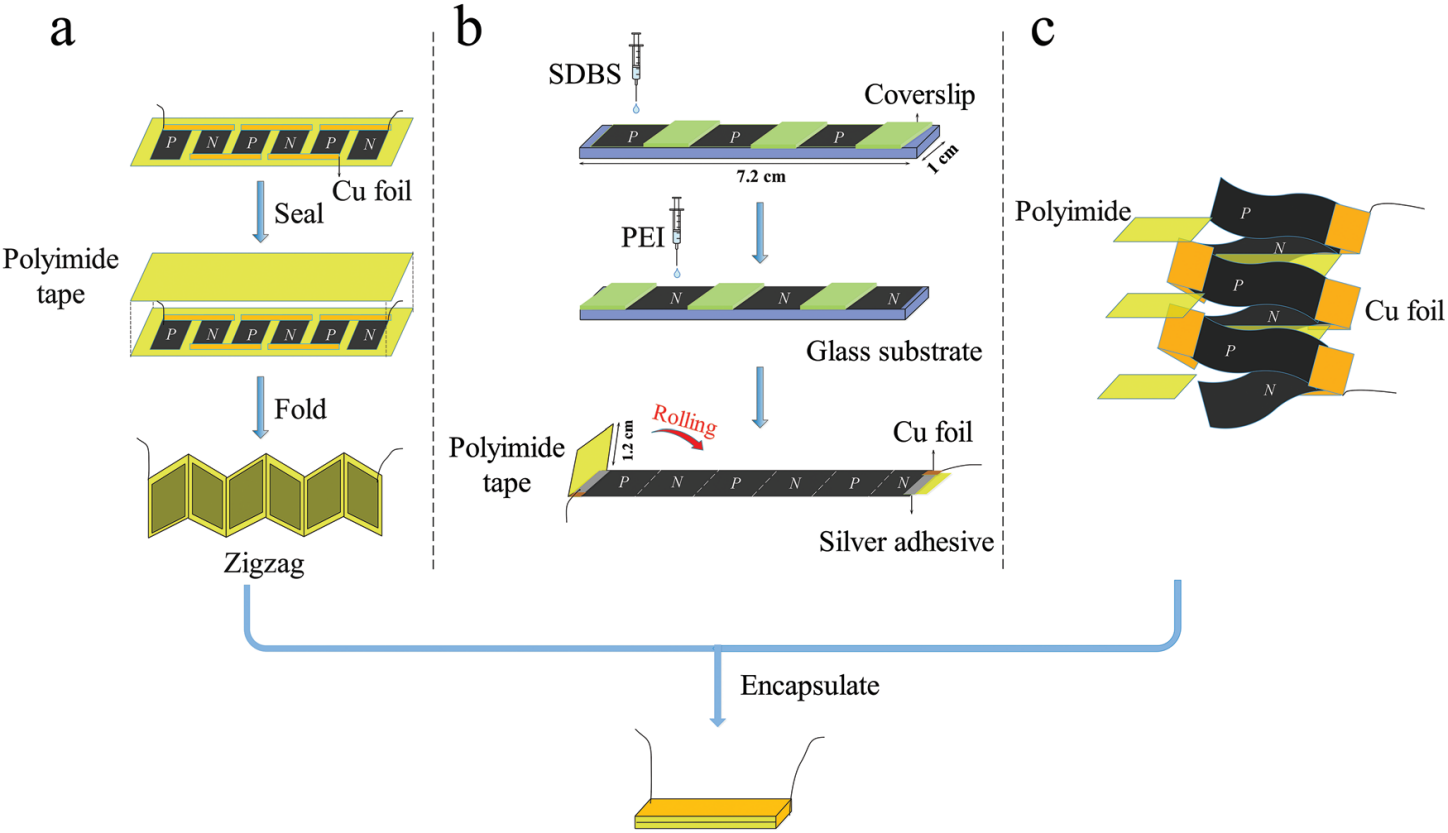
Schematic illustration of the three assembling configurations of flexible TE devices: a) serial, b) folding, and c) stacking devices.

**Figure** 3a schematically illustrates the experimental configuration for evaluating the performance of the TE device by using a home‐made apparatus, and a photograph of the real experimental setup is shown in Figure S7 (Supporting Information). In brief, the TE device was placed between a hot plate and a cold one. Two pairs of T‐type thermocouples were fixed to the hot and the cold ends of the device, respectively. The temperature of the device hot end was controlled by a temperature controller, while the cold‐end temperature was maintained at 300 K using a commercial Peltier module in contact with a circulating water cooler. The temperature difference across the device was kept constant (0–50 K) at which the device (b) open‐circuit voltage (*V*_AC_) and (c) output power (*P*_AC_) were measured. The theoretical open‐circuit voltage (*V*_TH_) can be predicted by

(2) $$V_{\mathrm{TH}}\;\;=\;\;N\left(S_{n}\;\;+\;\;S_{p}\right)\;\;\times \;\;\Delta T$$

where *S*_n_ and *S*_p_ are the Seebeck coefficients of the n‐ and p‐type TE films, respectively. Thus, the *V*_TH_ is defined by *N*, *S*_n_, *S*_p_, and Δ*T*. For a device composed of the same *N* and the p‐n couples with the same *S*_p_ and *S*_n_, *V*_TH_ increases with Δ*T*. Figure 3b clearly shows that unlike *V*_TH_, the device configuration and assembly modes greatly affect the device *V*_AC_. All of the *V*_AC_ increase with Δ*T*, and the *V*_AC_ for the stacking configuration enhance slowly. Furthermore, at the same Δ*T*, the voltages almost follow the sequence of theoretical > serial > folding > stacking, where the maximum *V*_AC_ is 4.6 mV at Δ*T* = 49 K for the serial device.

**Figure 3:**
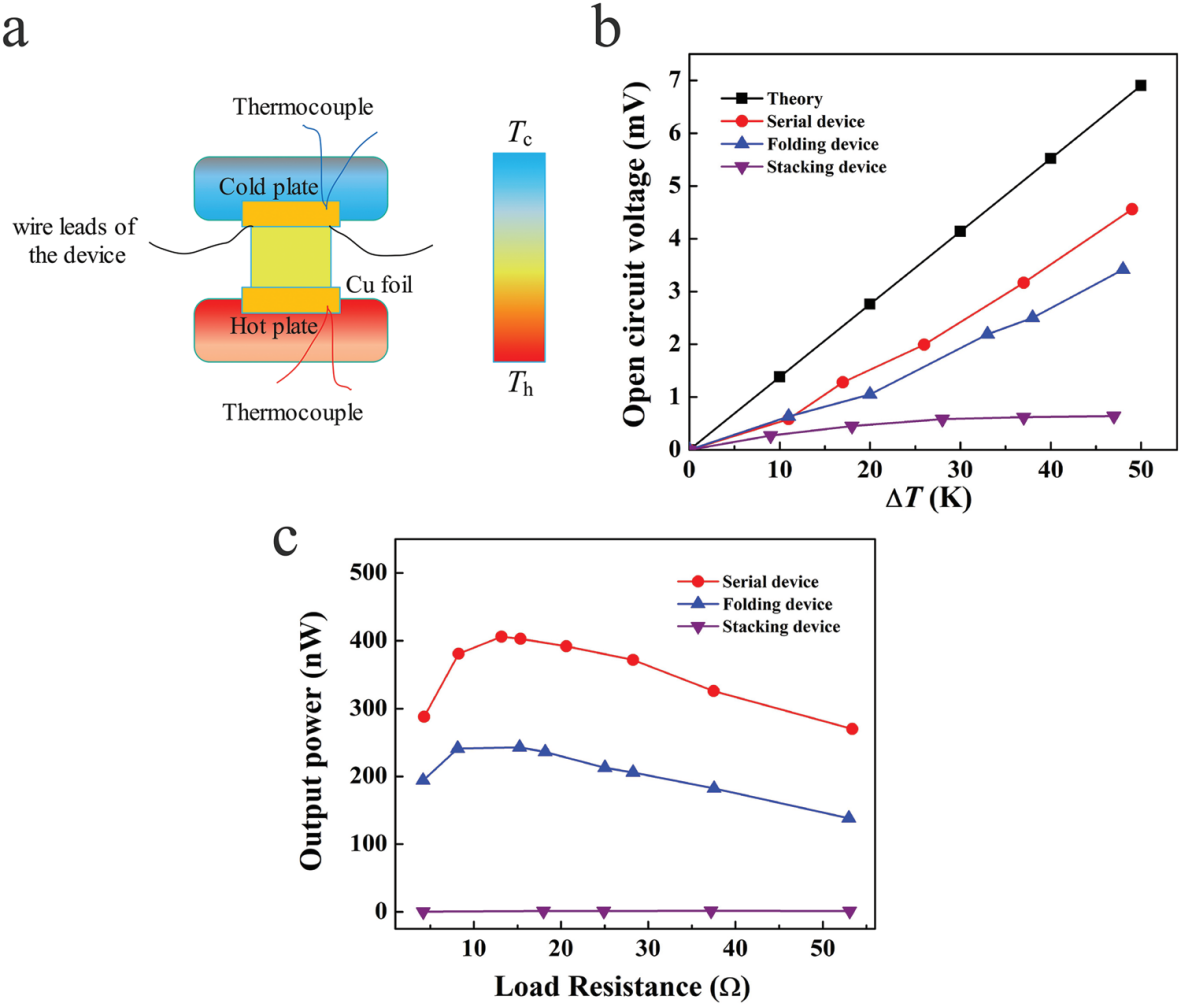
a) A schematic illustration of the TE generator to measure the device performance. The theoretical values and the measured data of the TE performances for the three types of TE devices: b) dependence of open‐circuit voltage with Δ*T*, and c) effect of load resistance on the output power.

The output power is vital for the practical applications of TE devices. The dependence of output power on *R*_L_ is described by the following equation

(3) $$P_{\mathrm{AC}}\;\;=\;\;{\left(\frac{V_{\mathrm{AC}}}{R_{r}+R_{L}}\right)}^{2}\;R_{L}\;\;=\;\;\frac{V_{\mathrm{AC}}{}^{2}}{\frac{{\left(R_{r}\;\;-\;R_{L}\right)}^{2}}{R_{L}}\;\;+\;\;4R_{r}}$$

where *R*_r_ and *R*_L_ represent the intrinsic internal and the load resistances, respectively. Thus, for a certain TE device at a constant Δ*T*, *P*_AC_ reaches its maximum when *R*_r_ equals *R*_L_. Figure 3c shows the output power as a function *R*_L_ at Δ*T* = 50 K. The maxima for the serial, the folding, and the stacking devices are 406 nW (*R*_r_ = 13 Ω), 243 nW (*R*_r_ = 15 Ω), and 1.56 nW (*R*_r_ = 37 Ω), respectively. Indeed, the junction between p‐type and n‐type films as well as the intercalation of electrically insulating polyimide slices will inevitably result in the significant increase of *R*_r_.

**Table** 1 illustrates a brief summarization of the performance for flexible TE devices, including some typical examples in previous literatures and the present work. Regretfully, although the output power has always been employed to characterize the device TE performance, no general parameter or method has ever been set up to effectively compare or evaluate the assembly strategies for flexible TE devices to date. Various influencing factors, such as the applied Δ*T*, the number of p‐n couples (*N*), and even the mass of the device (*m*) will remarkably affect the device TE performance. As a result, it is impossible to judge the device assembly configurations and compare the fabrication strategies from the results in the available publications. To solve this problem, here, we propose a convenient method to evaluate the FDP by dividing the maximum output power (*P*_max_) with the device mass (*m*), Δ*T*, and *N*, as described in Equation (1), that is, FDP $=\;\;\frac{P\max}{m\Delta TN}\;$. In the present study, the FDPs for the serial, folding, and stacking assembling configurations are 11.13, 8.87, and 0.05 nW g^−1^ K^−1^, respectively. Therefore, we conclude that the serial type is superior to the folding assembling configuration, and the stacking device is poor in conversion of heat to electricity. Indeed, Figure 3 also demonstrates that the device TE performances of both open‐circuit voltage (b) and output power (c) follow the same sequence of serial > folding ≫ stacking. Considering that the same materials of the p‐ and n‐type units, and the same number and size dimensions of the p‐n couples, it is reasonable to conclude that the serial assembly configuration exhibits the highest TE performance for flexible devices, the stacking type presents the lowest, while the folding mode is the medium in heat‐to‐electricity conversion capability. The reasons for the TE performance deviance may include the *R*_r_ difference resulting from the connection of p‐n units, the insulating layers, and the interfacial resistance between the p‐n couples with Cu foil or Ag top electrodes deposited on the TE legs.

**Table 1:** A summary of TE properties of the flexible devices

Assembly mode	Number of p‐n couples (*N*)	Mass (*m*) [g^−1^]	Temperature gradient (Δ*T*) [K]	Output power (*P*) [nW]	FDP [nW g^−1^ K^−1^]	Ref.
Serial	3 pairs	0.243	50	406	11.13	This work
Folding	3 pairs	0.183	50	243	8.87	This work
Stacking	3 pairs	0.224	50	1.56	0.05	This work
Serial	6 pairs	–	50	220	–	26
Serial	5 pairs	–	50	4.5	–	31
Serial	8 pairs	–	20	40.3	–	32
Serial	63 pairs	–	20	140	–	33
Stacking	72 pairs	–	32	1800	–	28
Stacking	14 pairs	–	55	649	–	30

In summary, we compare the fabrication strategies of flexible TE devices using three different assembly configurations, that is, serial, folding, and stacking, with the same p‐ and n‐type SWCNT TE couples. We put forward a convenient and general method to evaluate the TE performance for flexible devices, that is, $\mathrm{FDP}\;\;=\;\;\frac{P\max}{m\Delta TN}$. Although only CNT is employed in the present study, it is reasonable to use FDP to compare the assembly configurations for flexible devices made of other TE materials. Aided by the present data of 11.13, 8.87, and 0.05 nW g^−1^ K^−1^, we conclude that the serial mode is better than the folding one, while the stacking style is poor to realize heat‐to‐electricity conversion. We believe that our present study is an important trial in rational design of assembling strategy and performance evaluation of flexible TE devices, which will significantly benefit future in‐depth research and widen versatile applications of waste‐heat harvesting, e‐skin, wearable electronics, etc.

## Experimental Section

*Materials*: Commercialized SWCNTs with a diameter <3 nm and a purity >85.0 wt% were bought from Shenzhen Nanotech Port Co. Ltd., China. SDBS and branched PEI (molecular weight: 600, 99%) were purchased from Aladdin Company. All of the other regents, including nitric acid (HNO_3_), sulfuric acid (H_2_SO_4_), anhydrous ethanol (A.R.), and distilled water, were used as received without further purification.

*Preparation of Acid‐Doped SWCNT Films*: First, 200 mg of SWCNT was added into 60 mL of mixed acid solution (HNO_3_/H_2_SO_4_, 3:1 (v/v)). Then, the mixture was heated and refluxed at 80 °C for 4 h. Finally, the mixture was diluted, vacuum‐filtered on a porous nylon membrane, and dried under vacuum at 60 °C for 30 min, providing a SWCNT bucky paper (10 cm in diameter, ≈41 µm in thickness).

*Fabrication of Serial Device*: The acid‐doped SWCNT films were cut into six rectangular ribbons with dimensions of 11 × 10 mm^2^. Three of them were drop‐casted by 1 wt% SDBS aqueous solution and dried under vacuum at 80 °C for 30 min, resulting in p‐type unit films. Similarly, the other three SWCNT ribbons were drop‐casted by 1 wt% PEI aqueous solution and dried under vacuum at 80 °C for 30 min, affording n‐type films. As shown in Figure 2a, three pairs of p‐ and n‐type SWCNT films were first alternatively connected in series using copper foil and sliver electrodes on a polyimide substrate. Then, they were sealed by polyimide tape for protection to avoid air contact and oxygen doping. Subsequently, the device was folded in zigzag shape and encapsulated by polyimide tape. Finally, two copper wires were embedded into the cold end for measuring the output voltage.

*Fabrication of Folding Device*: The procedure is schematically shown in Figure 2b. First, the acid‐doped SWCNT bucky paper was cut into a long strip (72 × 10 mm^2^), placed on a glass substrate, and covered by three coverslips (12 × 11 mm^2^) at the same intervals. Then, the parts without coverslips were drop‐casted by 1 wt% SDBS aqueous solution (≈200 µL) with a micropipette, and dried under vacuum at 80 °C for 30 min. Similarly, the other parts were drop‐casted and doped with 1 wt% PEI aqueous solution, by changing the coverslips from the original positions to the SDBS‐SWCNT sites. Thus, a continuous film made up of three pairs of p‐ and n‐type units was obtained. The dimensions of the p‐ and n‐type units (11 mm × 10 mm × 41 µm) were the same with the above serial devices. After that, the film was transferred onto a polyimide tape substrate (90 × 11 mm^2^), where a blank part (12 × 11 mm^2^) in the left end was used as an insulator layer. Finally, the polyimide tape was rolled in clockwise direction. Two copper wires were embedded into the device cold end.

*Fabrication of Stacking Device*: The preparation of the p‐ and n‐type SWCNT films was the same as the above serial device. As displayed in Figure 2c, three pairs of the p‐ and n‐type SWCNT films were electrically connected by the way of multilayered stacking with copper foil and sliver paste. Polyimide films (≈40 µm) were inserted among adjacent p‐ and n‐type films to avoid any electrical contact. Finally, the stacking device was encapsulated by polyimide tape for protection. Similarly, two copper wires were embedded into the cold end of TE device.

*Morphology and Structural Characterizations*: The surface morphology was directly observed using a HITACHI S‐4800 scanning electron microscope at an acceleration voltage of 15 kV. Raman spectra were recorded within the wavenumber range of 500–3100 cm^−1^ with a Raman spectrometer (Lab‐RAM HR Evolution) using a laser diode at an excitation wavelength of 532 nm. XPS analysis was conducted by a multipurpose X‐ray photoemission spectroscopy (Thermo Scientific ESCALAB 250Xi). FTIR spectra were collected with a Perkin‐Elmer System 2000 FTIR spectrophotometer with 32 scans in the wavenumber range of 4000–400 cm^−1^.

*Measurements of TE Performance*: The electrical conductivities and the Seebeck coefficients at room temperature were measured by a commercial instrument, Film Thermoelectric Parameter Test System (MRS‐3), JiaYiTong Company. During the measurements, a quasi‐steady state mode was adopted. The resistances and the output voltages of the TE devices were measured by Keithley 192 Programmable DMM and a Keithley 2000 Multimeter (Keithley Instruments Inc., USA), respectively.

The TE performance of the flexible devices was measured schematically shown in Figure 3a of the experimental configuration by a home‐made apparatus. As evidently displayed in Figure S7 (Supporting Information), the experimental apparatus consists of a hot plate, a cold plate, temperature control systems, and a Keithley 2400 sourcemeter. First, after the TE device was placed between a hot plate and a cold plate the two pairs of T‐type thermocouples were fixed to the either sides of the device. The temperature of the device hot end was controlled by a temperature controller (Lakeshore 336), and the temperature of the cold end was maintained at 300 K using a commercial Peltier module contacting with a circulating water cooler. The temperature difference (Δ*T*) across the device was maintained at desired values between 0 and 50 K, while the output power (*P*) and the open‐circuit voltage (Δ*V*) of the device were monitored. For measuring the *P*, the device was connected in series with a load resistor (*R*_L_), and the Keithley 2400 sourcemeter was configured to measure electrical current (*I*_C_) in the circuit. Then, the output power was calculated from the expression *P* = *I*_c_^2^ *R*_L_. In order to optimize the maximum output power, the generated current (Figure S8, Supporting Information) and load circuit voltage (Figure S9, Supporting Information) from the flexible TE devices were measured. In the measurements of the open‐circuit voltage (*V*_AC_), the load resistor was removed, and the TE device was directly connected to the Keithley 2400 sourcemeter.

## Conflict of Interest

The authors declare no conflict of interest.

## Supporting information

Supplementary
