# Supplementary material for: Assembly Strategy and Performance Evaluation of Flexible Thermoelectric Devices
Source: Adv Sci (Weinh). 2019 May 22;6(15):1900584. doi: 10.1002/advs.201900584 (PMC6685505; doi:10.1002/advs.201900584)
Supplement: Supplementary file 1 — Supplementary [file ADVS-6-1900584-s001.pdf]

Copyright WILEY-VCH Verlag GmbH & Co. KGaA, 69469 Weinheim, Germany, 2019.

Supporting Information

for *Adv. Sci.,* DOI: 10.1002/advs.201900584

Assembly Strategy and Performance Evaluation of Flexible Thermoelectric Devices

Dawei Qu, Xin Li, Hanfu Wang,* and Guangming Chen**

Copyright WILEY-VCH Verlag GmbH & Co. KGaA, 69469 Weinheim, Germany, 2019.

Supporting Information

Assembly Strategy and Performance Evaluation of Flexible Thermoelectric Devices

Dawei Qu, Xin Li, Hanfu Wang,* and Guangming Chen**

D. Qu, Prof. G. Chen
College of Materials Science and Engineering, Shenzhen University, Shenzhen 518055, P. R. China
E-mail: chengm@szu.edu.cn (G. Chen)
D. Qu, Prof. X. Li
Beijing Key Laboratory of Clothing Materials R&D and Assessments, Beijing Engineering Research Center of Textile Nanofiber, School of Materials Science & Engineering, Beijing Institute of Fashion Technology, Beijing 100029, P. R. China
E-mail: clylx@bift.edu.cn
Prof. H. Wang
National Center for Nanoscience and Technology of China, Beijing 100190, P. R. China
E-mail: wanghf@nanoctr.cn

Table of Contents

Optical image showing the high flexibility of acid-doped SWCNT film 2
FESEM images of 5 wt% SDBS or PEI doped SWCNTs 3
Raman spectra and radial breathing mode (RMB) profiles for the SWCNTs 4
FTIR spectra of the SWCNTs 5
Photographs of the three types of TE device configurations 6
Photograph showing the flexibility of the TE device. 7
Photograph of the TE generator to measure the device performance 8
Electrical current as a function of load resistance at various ΔT 9
Load circuit voltage as a function of load resistance at various ΔT 10

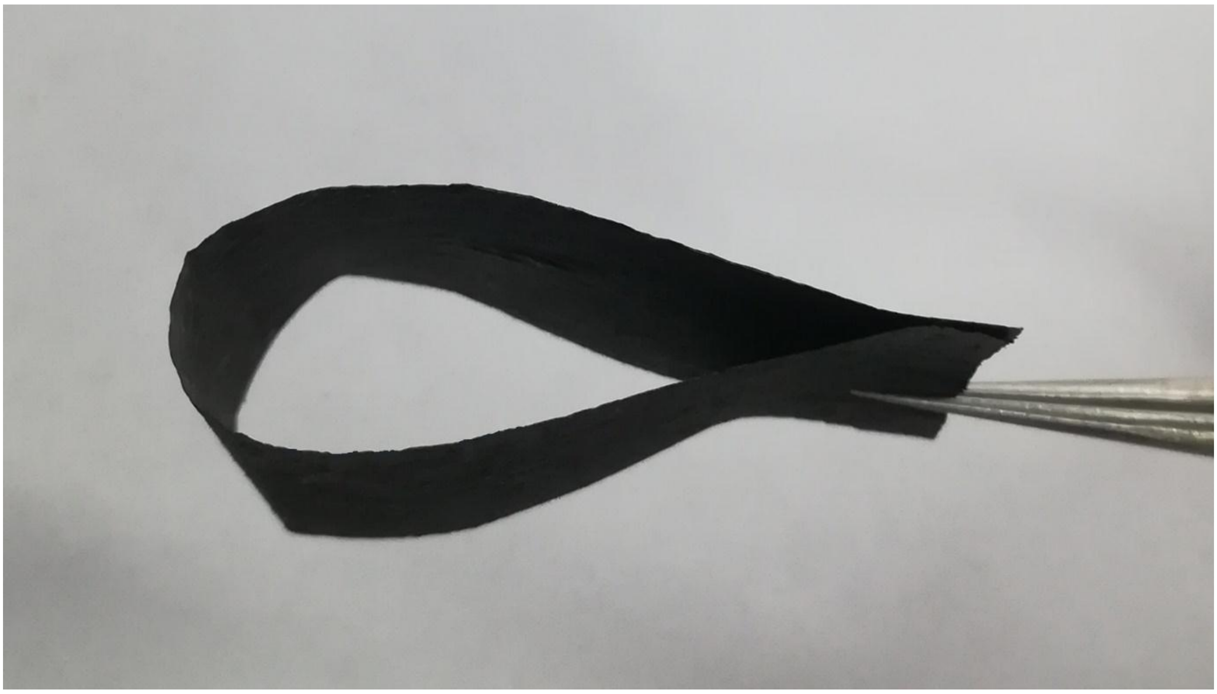

Figure S1. Optical image showing the high flexibility of the acid-doped SWCNT film before the p- and n-type treatments.

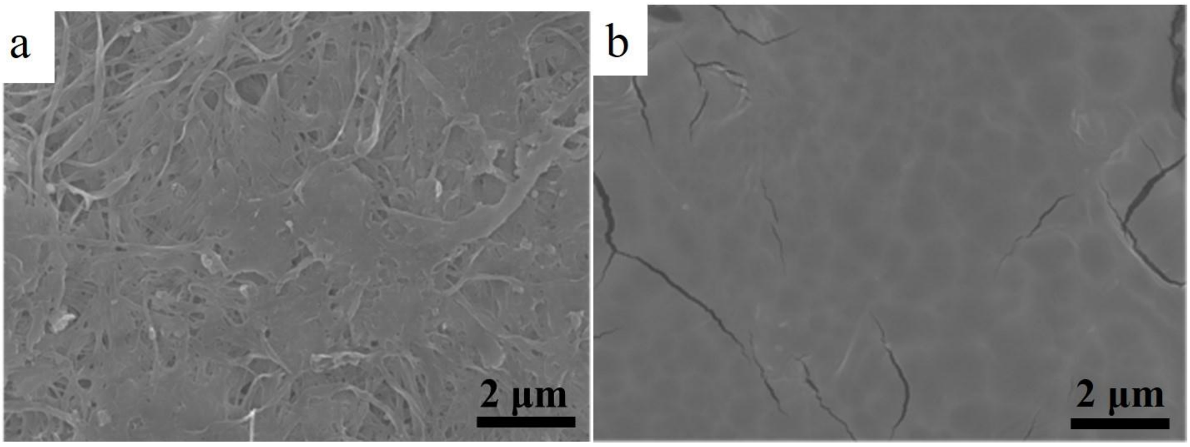

Figure S2. FESEM images of surface morphology for the flexible SWCNT films chemically doped by 5 wt% SDBS and PEI aqueous solutions, respectively.

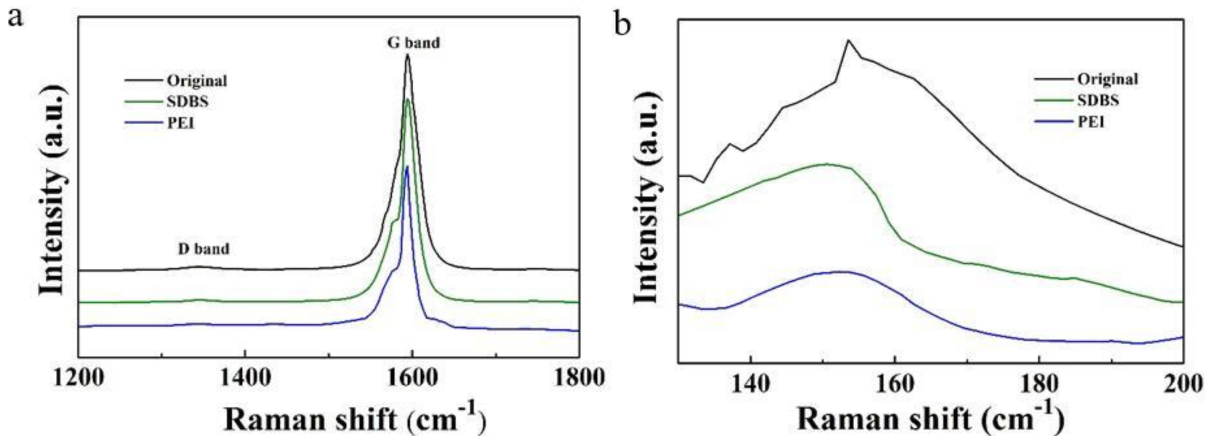

Figure S3. (a) Raman spectra and (b) radial breathing mode (RMB) profiles of the pristine acid-doped SWCNT, and the SDBS or PEI doped SWCNT using 1 wt% organic doping agent.

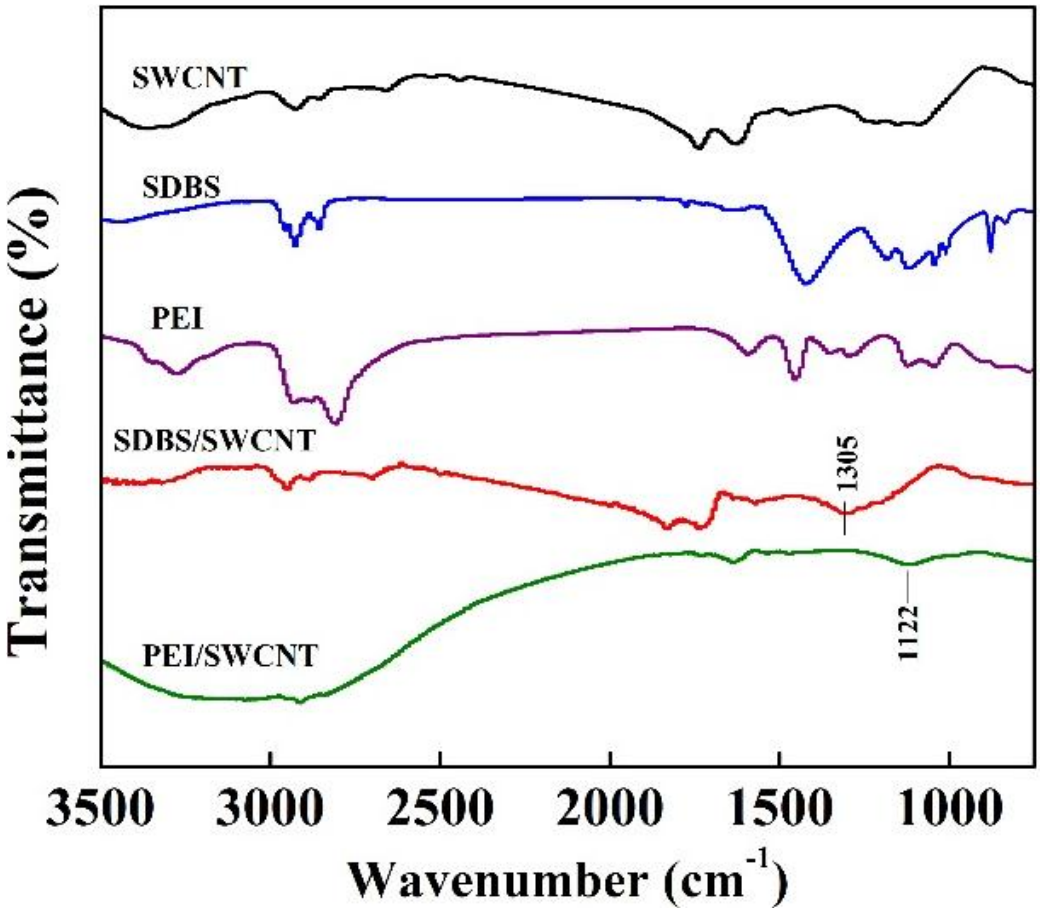

Figure S4. FTIR spectra of the acidified SWCNT, SDBS, PEI, and the p- or n-type chemically doped SWCNT by SDBS or PEI.

In the spectra of acidified SWCNT, the bands at 2929 cm^{-1}, 1731 cm^{-1} and 1622 cm^{-1} are due to C-H, C=O, C=C, stretching vibration modes, respectively. The characteristic bands for S-O stretching vibration modes of SDBS ranges from 1250 to 1100 cm^{-1}. And the bands between 1230 and 1030 cm^{-1} result from the C-N stretching vibration modes of PEI. Due to the interfacial interaction with SWCNT, the bands at 1305 cm^{-1} and 1122 cm^{-1} characteristic of SDBS and PEI, respectively, become broadened for the SDBS or PEI doped SWCNT.

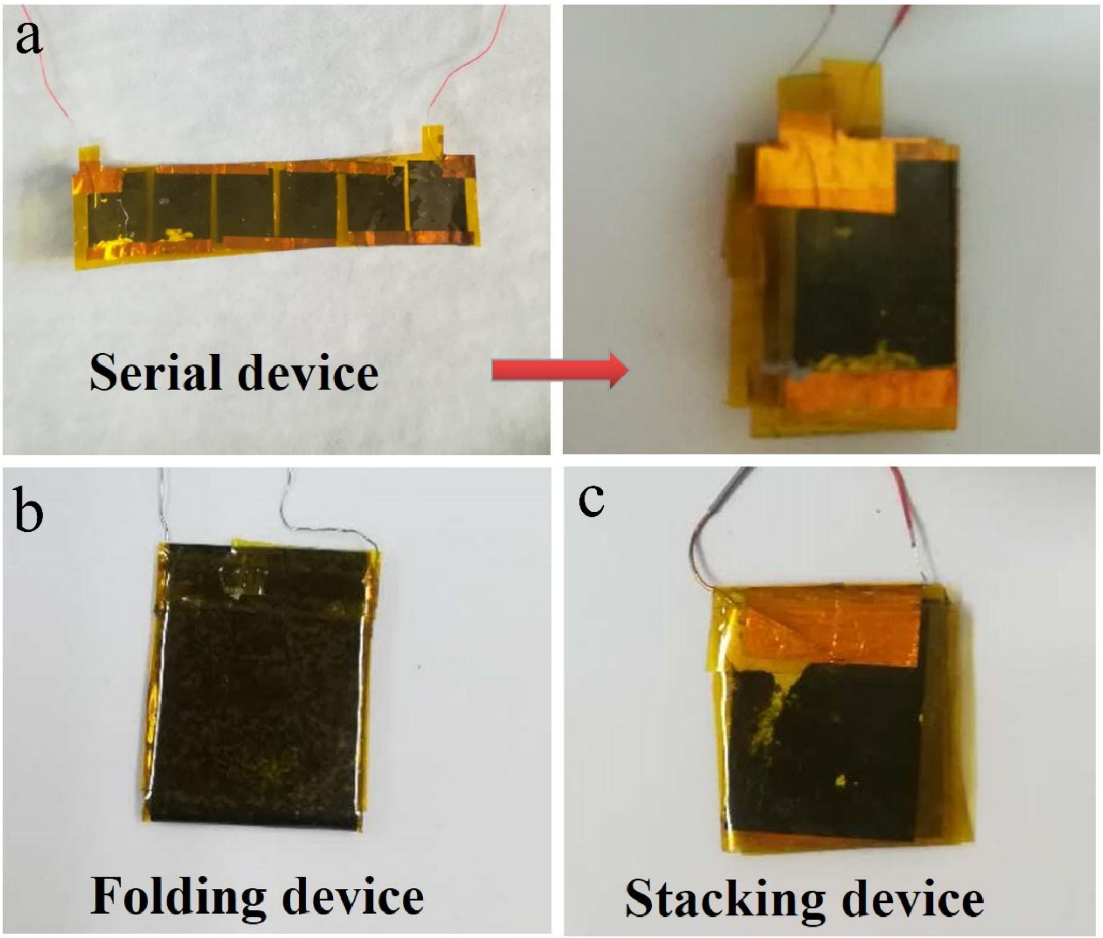

Figure S5. Photographs of the three types of TE device configurations, *i.e.* (a) serial, (b) folding, and (c) stacking devices.

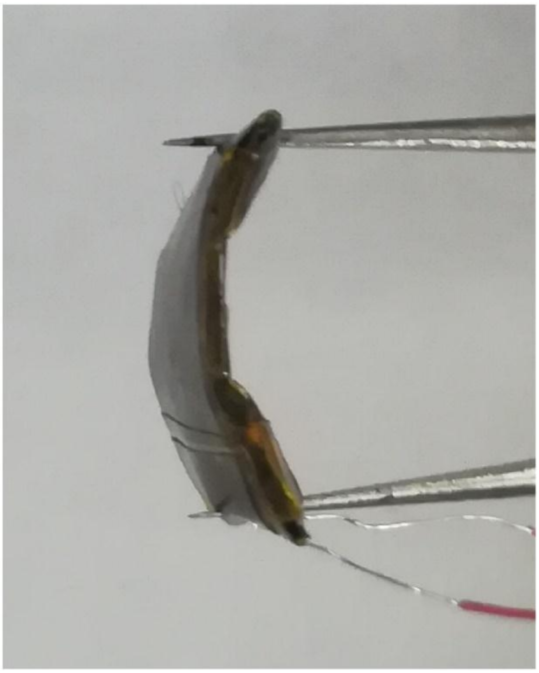

Figure S6. Photograph showing the flexibility of the TE device.

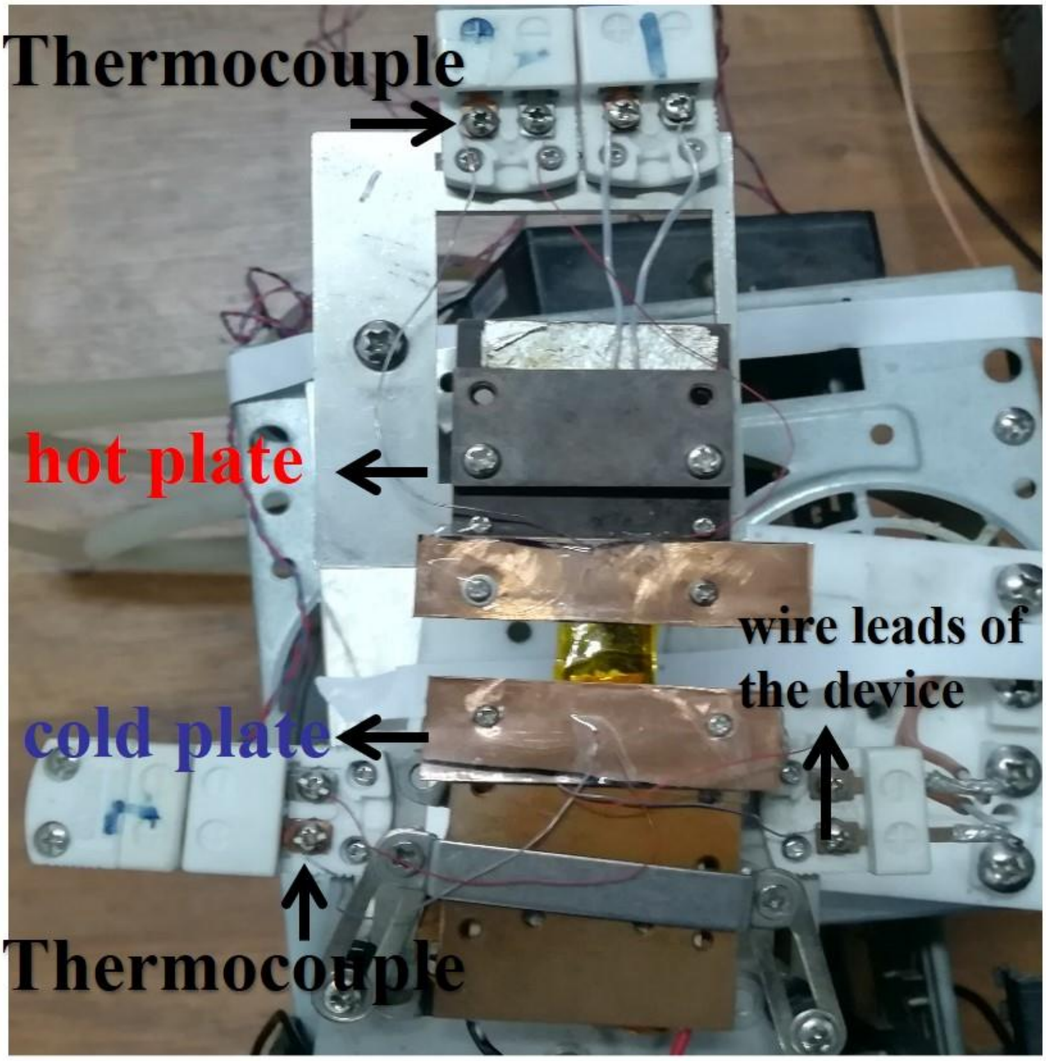

Figure S7. Photograph of the TE generator to measure the device performance.

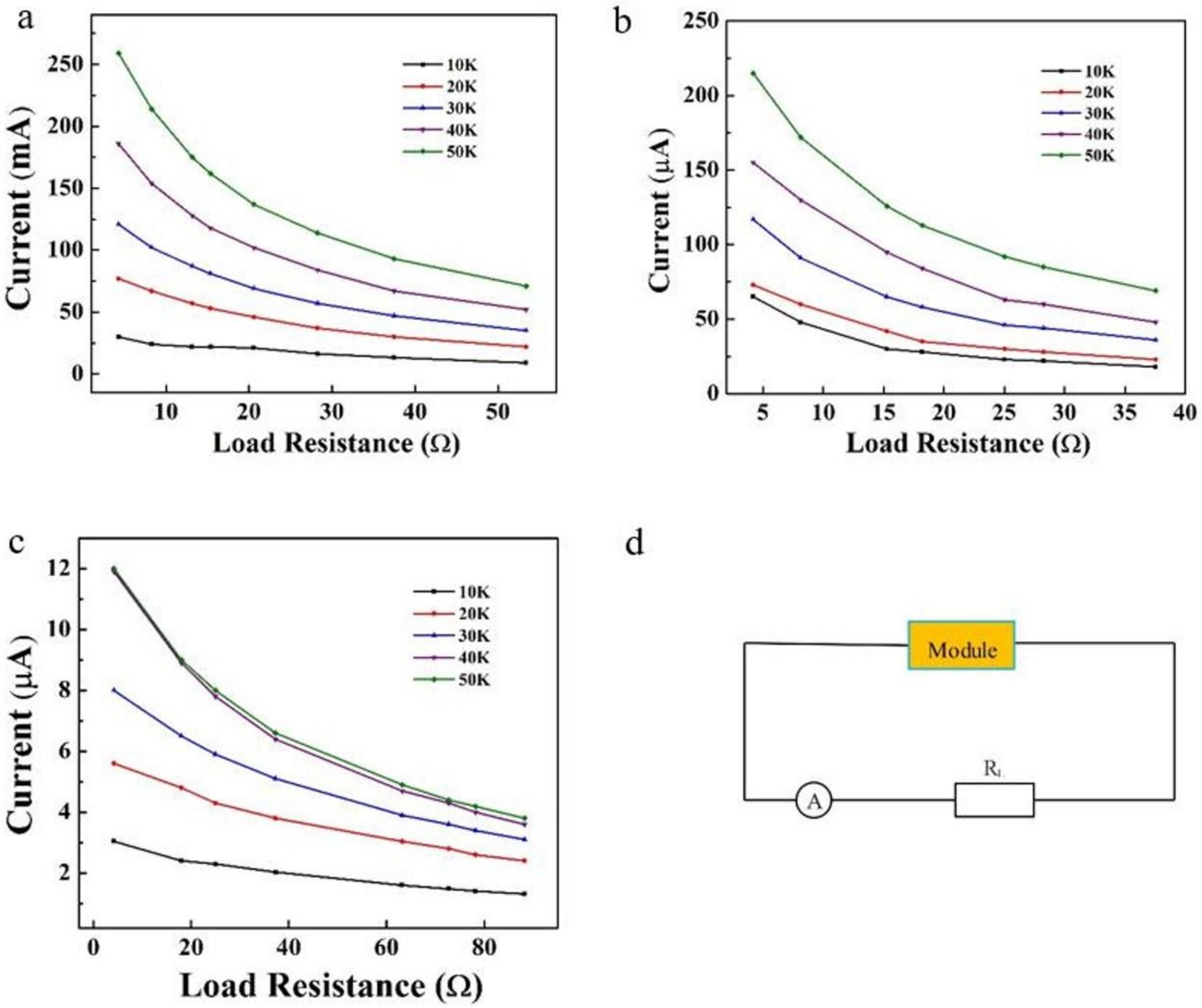

Figure S8. The electrical current of the TE devices with the three assembly configurations of (a) serial, (b) folding and (c) stacking, as a function of load resistance at temperature gradients (ΔT) of 10–50 K. (d) A schematic of the circuit flow chart.

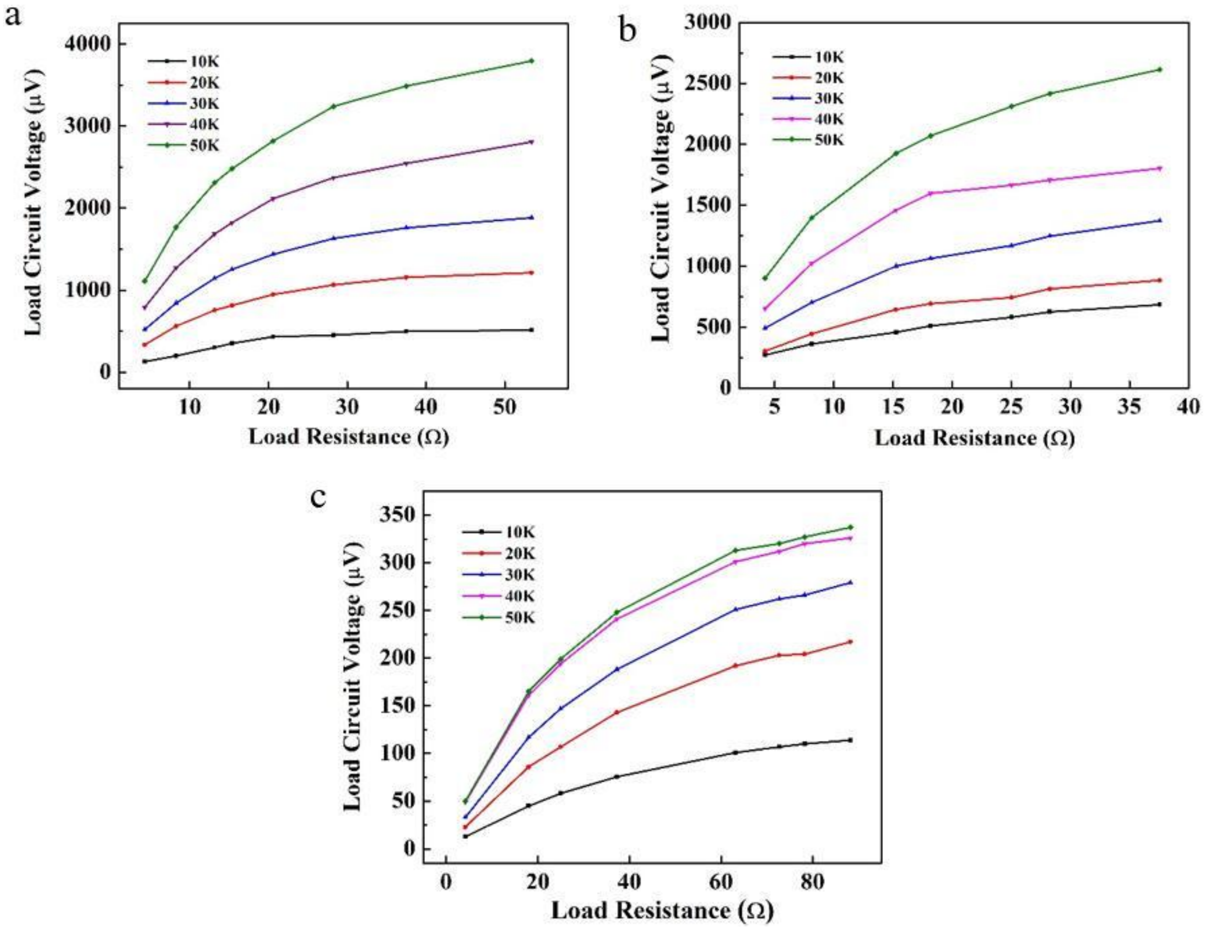

Figure S9. The load circuit voltage of the three types of TE devices, (a) serial, (b) folding and (c) stacking devices, as a function of load resistance at temperature gradients (ΔT) of 10–50 K.
